# Multiple Beneficial Effects of Ghrelin Agonist, HM01 on Homeostasis Alterations in 6-Hydroxydopamine Model of Parkinson’s Disease in Male Rats

**DOI:** 10.3389/fnint.2019.00013

**Published:** 2019-04-12

**Authors:** Artem Minalyan, Lilit Gabrielyan, Claudio Pietra, Yvette Taché, Lixin Wang

**Affiliations:** ^1^Veterans Affairs Greater Los Angeles Healthcare System, Los Angeles, CA, United States; ^2^Helsinn SA Lugano, Lugano, Switzerland; ^3^CURE/Digestive Diseases Research Center, Digestive Diseases Division, Department of Medicine, David Geffen School of Medicine, University of California, Los Angeles, Los Angeles, CA, United States

**Keywords:** body composition, defecation, ghrelin agonist, 6-hydroxydopamine, rat, water intake

## Abstract

**Background and objective**: Developing therapy for non-motor symptoms of Parkinson’s disease (PD) is important for improving patients’ quality of life. Previously, we reported that the ghrelin receptor agonist, HM01 normalized the decreased 4-h fecal output and levodopa-inhibited gastric emptying in 6-OHDA rats, and activated selective areas in brain and spinal cord. In this study, we evaluated whether chronic HM01 treatment influences motor functions and/or has beneficial effects on non-motor symptoms including alterations of body weight and composition, defecation, feeding and water intake in 6-OHDA rats.

**Methods**: Male rats were microinjected unilaterally into the medial forebrain bundle with either vehicle or 6-OHDA. Three weeks later, we assessed basal body weight, and 24-h fecal output (pellets, weight, dry weight and water content), water intake and food intake (ingested and spillage). Then, HM01 (3 mg/kg) or vehicle was given per gavage daily for 10–12 days and the same parameters were re-assessed daily. Motor behavior (stepping and rotations tests), body composition were monitored before and after the HM01 treatment.

**Results**: 6-OHDA rats showed motor deficits in rotation test induced by apomorphine and stepping test. They also displayed a significant reduction in body weight, water consumption, fecal weight and water content and an increase in food spillage compared to vehicle microinjected rats. Daily oral treatment of HM01 did not modify motor alterations compared to vehicle but significantly increased the body weight, fat mass, and 24-h fecal weight, fecal water content, food and water intake in 6-OHDA rats, while HM01 had no significant effect in vehicle microinjected rats. Fecal weight and water content were both correlated with water intake, but not with food intake. Fat mass, but not body weight, was correlated with food intake. HM01 effects were significant after 24 h and remained similar during the treatment.

**Conclusions**: Chronic treatment with ghrelin agonist, HM01 improved several non-motor symptoms in the rat PD model induced by 6-OHDA lesion including the decrease in body weight, water consumption, fecal weight and water content, and increased food intake while not improving the motor deficits. These findings provide pre-clinical evidence of potential benefits of ghrelin agonists to alleviate non-motor symptoms in PD patients.

## Introduction

Parkinson’s disease (PD), although principally characterized by tremor and other motor impairments, also encompasses non-motor symptoms with alterations notably in the digestive system and body weight maintenance (Jost, 2010; Sakakibara et al., 2011). Constipation occurs with a high incidence at all stages (Stirpe et al., 2016; Knudsen et al., 2017) and the majority of PD patients also display body weight loss with a prominent decrease in fat mass (Lorefält et al., 2004; Sharma and Lewis, 2017). PD patients also showed reduced water intake compared to healthy controls (Ueki and Otsuka, 2004; Barichella et al., 2017). This adipsia is of potential relevance since two clinical surveys in PD patients pointed to an association between daily liquid intake and constipation (Ueki and Otsuka, 2004; Gan et al., 2018).

None of the animal models can replicate all of the PD pathological alterations and progression occurring in patients, however, each model recapitulates particular features that can have translational values (Beal, 2010; Bové and Perier, 2012; Blesa and Przedborski, 2014; Francardo, 2018). The model in which the neurotoxin, 6-hydroxyldopamine (6-OHDA) is microinjected into the substantia nigra (SN)-striatum pathway, has been widely used in preclinical studies to assess new compounds and largely contributed to drug development for the dopamine (DA) treatment of PD patients (Bové and Perier, 2012; Francardo, 2018). The 6-OHDA model is also relevant to study the impact of altered central DA signaling circuits on gastrointestinal (GI) function and other non-motor deficits occurring during PD progression (Karasawa et al., 2014a; Toti and Travagli, 2014). In particular, rats microinjected unilaterally with 6-OHDA into the medial forebrain bundle (mfb, containing the axons of nigrostriatal dopaminergic neurons) display decreased defecation, and reduced colonic contractions, water intake and body weight (Blandini et al., 2009; Colucci et al., 2012; Karasawa et al., 2014a; Fornai et al., 2016).

Ghrelin is a well-known pleiotropic gut hormone that regulates energy balance *via* enhancing appetite and adiposity, stimulates GI transit, and plays a role in rewarding behavior (Müller and Tschöp, 2013; Müller et al., 2015). Ghrelin actions are mediated by interaction with the growth hormone (GH) secretagogue receptor (GHS-R1a, or ghrelin receptor), a G protein-coupled receptor distributed in both in the peripheral and central nervous systems (Poitras and Tomasetto, 2009; Sallam and Chen, 2010; Müller and Tschöp, 2013; Müller et al., 2015). In particular, ghrelin receptors are expressed in SN neurons immunoreactive for tyrosine hydrolase (TH; Guan et al., 1997; Zigman et al., 2006; Jiang et al., 2008; Andrews et al., 2009) and are downregulated at this site in a mouse model of PD with motor dysfunction (Suda et al., 2018). Other studies indicate that ghrelin modulates dopaminergic neurons in the ventral tegmental area (VTA) and SN (Stievenard et al., 2017) and induces a neuroprotective effect in animal models of PD (Bayliss and Andrews, 2013; de Candia and Matarese, 2018; Morgan et al., 2018). In addition, clinical studies indicate that systemic or oral administration of ghrelin agonists increases appetite and body mass in patients with cancer cachexia (Argilés et al., 2017; Khatib et al., 2018). This supports a potential beneficial effect to improve nutritional and metabolic status of PD patients who have loss of appetite and body weight and reduced circulating ghrelin levels during the postprandial recuperation phase (Unger et al., 2011; Song et al., 2017). It is also of relevance that some ghrelin receptor agonists have moved to clinical trials for gastroparesis and constipation (Acosta et al., 2015; Mulak and Bonaz, 2015; Shin and Wo, 2015; Mosińska et al., 2017). Collectively, these findings suggest that ghrelin agonists may be of benefit to alleviate PD symptoms (Ramprasad et al., 2018).

In the present study, we investigated whether repeated treatment with the new orally active, long acting and blood brain barrier penetrant ghrelin agonist, HM01 (Karasawa et al., 2014a), had beneficial effects on several homeostatic dysfunctions in the 6-OHDA rat PD model not treated with L-dopa. We assessed simultaneously the alterations of feeding and drinking behavior, defecation, and body weight and composition induced by 6-OHDA and the influence of 10–12 days oral administration of HM01. In addition, we examined whether HM01 influenced 6-OHDA altered motor functions in the stepping and rotation tests.

## Materials and Methods

### Animals

Adult male Sprague-Dawley (SD) rats (250–270 g, Harlan Laboratories, San Diego, CA, USA) were kept under controlled illumination (12:12 h light/dark cycle, lights on/off: 6:00 AM/6:00 PM) and temperature (22 ± 2°C). Animals were fed a standard rodent diet (Prolab RMH 2,500; LabDiet, PMI Nutrition, Brentwood, MO, USA) and tap water *ad libitum*. Animal care and experimental procedures followed institutional ethic guidelines and conformed to the requirements of federal regulations for animal research conduct. All procedures were approved by the Animal Research Committee at Veterans Affairs Greater Los Angeles Healthcare System (animal protocol #01001–15). The experiments were performed in non-fasted rats.

### Reagents

The ghrelin agonist, HM01 (provided by Helsinn SA Lugano, Lugano, Switzerland) was suspended in vehicle (0.5% carboxymethyl cellulose, CMC), except otherwise stated. The neurotoxin, 6-OHDA hydrochloride (Sigma-Aldrich Co., St. Louis, MO, USA) was dissolved in a saline solution containing 0.2% ascorbic acid. Apomorphine (Sigma-Aldrich Co., St. Louis, MO, USA) was dissolved in saline.

### 6-OHDA Rat Model of PD

The procedure was as described in our previous study (Karasawa et al., 2014a) and similar as reported by others (Blandini et al., 2009; Decressac et al., 2012; Gambaryan et al., 2014; Pellegrini et al., 2017). In brief, rats were anesthetized with an intramuscular injection of ketamine hydrochloride (75 mg/kg, Ketanest; Fort Dodge Laboratories, Fort Dodge, IA, USA) and xylazine (5 mg/kg, Rompun; Mobay Corporation, Shawnee, KS, USA), and placed on a stereotaxic apparatus (David Kopf Instruments, Tujunga, CA, USA). Then, 6-OHDA (12 μg in 3 μl) or vehicle (3 μl saline with 0.2% ascorbic acid) was microinjected unilaterally into the mfb using the following coordinates (mm) from the bregma (anterior-posterior: −4.4; medial-lateral: +1.2; and dorsal-ventral: −8.0) according to Paxinos and Watson (2007) atlas. Thereafter, rats were housed individually and experiments started after a 3-week period to reach a stabilized degeneration of the nigrostriatal pathways (Bové and Perier, 2012). The localizations of microinjection sites were identified by the needle trail in brain sections under microscopic examination and by immunohistochemistry for tyrosine hydroxylase (TH) at the end of each protocol as in previous studies (Wang et al., 2009).

### Measurements

#### Food and Water Intake, and Fecal Output

Rats housed individually were given pre-weighed food and water bottle with a ballpoint sipper tube in order to avoid water spillage as described previously (Karasawa et al., 2014a,b). Twenty-four hours later, water bottle was weighed, food remained in the feeder were weighed together with small pieces of chow dropped in the bedding, and the differences were calculated. The fecal pellets were counted, weighed, dried in an oven for 24 h and reweighed. The fecal water content was calculated by subtracting dry weight from wet weight.

#### Food Spills

Rats were housed individually in cages with a wire grid at the bottom and given pre-weighed food and water bottle. Twenty-four hours later, food remained in the feeder and food spills under the wire grid were weighed separately. Food intake was calculated by subtracting the food remained in the feeder and spills from pre-weight.

#### Body Composition

It was measured using a rodent magnetic resonance imaging (MRI) body composition analyzer (EchoMRI 700, EchoMedical Systems, Houston, TX, USA) as in our previous studies (Stengel et al., 2013). Changes in fat and lean mass and body water content were calculated relative to the values before HM01 treatment.

#### Motor Impairment Tests

The methods were adapted from previous publications (Olsson et al., 1995; Mehta et al., 2005; Decressac et al., 2012). The tests were video-recorded and two observers blind to experimental conditions counted the motor behaviors. Stepping test: rats were trained for the procedure 2 days before the test. Rats were held by the experimenter with one hand fixing the hindlimbs and right forelimb and slightly raising them above the surface of the table while the left forelimb (the impaired limb by opposite site with the 6-OHDA lesion that is rostral to pyramidal decussation) was unrestrained and touched a table. Rats were moved sideways 90 cm in 5 s on the table surface and the number of adjusted steps were counted. The procedure was repeated three times and the average was calculated. Rotation test: rats were injected intraperitoneally (ip) with apomorphine (0.5 mg/ml/kg) and placed in an empty cage and videotaped for 30 min. Rotations that usually start within a few min post-injection, were counted for 15 min and the number of rotations per min were calculated for each rat.

### Experimental Protocols

**Figure 1:**
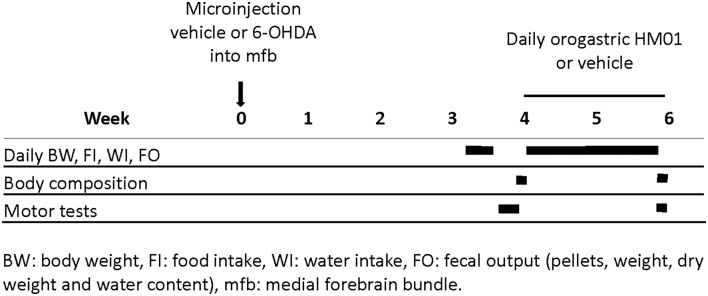
Schematic illustration of experimental protocols time course and related determinations.

Schematic representation of protocols in the Figure 1 indicates the timelines of different treatments and measurements during the experimental period. Different cohorts of rats were used for each experiment.

#### Effects of Daily HM01 Treatment on Body Weight and Composition, 24-h Food, Water and Fecal Measurements in 6-OHDA Rats

Rats were microinjected with 6-OHDA or vehicle (control), and 3 weeks after, basal 24-h food and water intake, fecal output (number, fecal dry weight and water content) and body weight were measured for 2–3 days. Thereafter, rats received daily og administration of HM01 (3 mg/kg) or vehicle between 9–10 AM for 10–12 days. Body weight, food and water intake, and defecation were monitored every day at 24 h intervals. Body composition was measured before and at the end of HM01 treatments. The 3 mg/kg dose selected was based on our previous dose-response study showing a maximal increase in the 4-h fecal weight induced by og HM01 administration in rats (Karasawa et al., 2014a).

#### Food Spills

Three weeks after vehicle or 6-OHDA microinjections, rats were acclimated to the housing conditions of a wire grid floor for 2 days. On the experiment day, rats were given pre-weighed food and 24 h later, food and food spills under the wire grid were weighed.

#### Effect of HM01 on Motor Functions in 6-OHDA Rats

Rats were microinjected with 6-OHDA or vehicle, and 3 weeks later they received the daily gavage with HM01 (3 mg/kg). The motor tests (stepping and rotation tests) were performed 1–3 days before and on the 11th (stepping) and 12th day (rotation) at 4–6 h after the last og HM01 treatment (3 mg/kg).

### Data Analysis and Statistics

Data are presented as mean ± standard error of the mean (SEM). The 24-h food and water intake and pellet output were calculated per 300 g body weight and the 10-day treatment period with HM01 on these parameters was expressed as 24-h averages of daily measurements. Statistical analysis was performed using SigmaPlot 12.5 (Systat Software, Inc., San Jose, CA, USA). Comparisons between two groups were performed by the Student’s *t*-test and among multiple groups by one-way or two-way analysis of variance (ANOVA) followed by Tukey *post hoc* multiple comparisons. Time course of HM01 stimulatory effects on daily feeding, drinking and defecation for the 10 days treatment were analyzed by repeated measures one-way ANOVA followed by Tukey test for all pairwise multiple comparisons. Correlations were performed by a Lineal Regression. A *p*-value < 0.05 was considered as significant.

## Results

### Chronic Daily HM01 Treatment did Not Improve Motor Deficits in Rats With Unilateral Lesion of Medial Forebrain Bundle Induced by 6-OHDA

**Figure 2:**
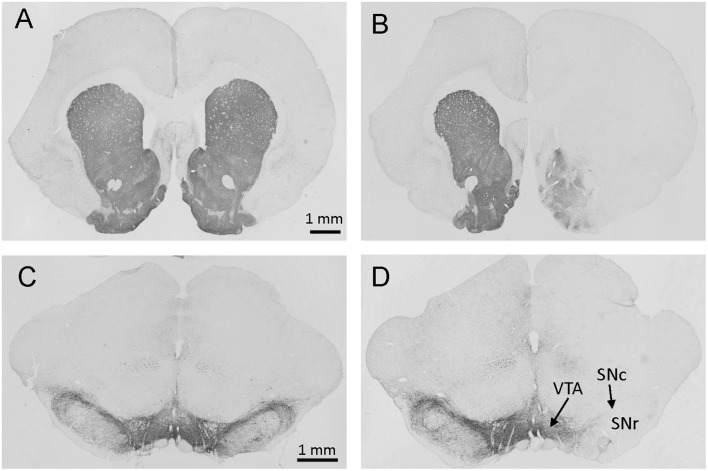
Photomicrographs of brain sections with tyrosine hydroxylase (TH) immunostaining at levels of the striatum **(A,B)** and substantia nigra (SN; **C,D**) from rats microinjected with vehicle **(A,C)** or 6-OHDA **(B,D)** in the right site of medial forebrain bundle (mfb). The immunostaining was processed about 6 weeks after mfb microinjection. Marked reductions in striatal fiber density **(B)** and nigral DA neurons **(D)** were shown on the side of 6-OHDA injection (right). SNc: substantia nigra pars compacta; SNr: SN pars reticulata; VTA: ventral tegmental area.

Rats microinjected unilaterally into the mfb with 6-OHDA (6-OHDA rats) lost the majority of TH-immunoreactive neurons in the SN and fibers in the striatum ipsilateral to the lesion site observed about 6 weeks later at the end of experiments (Figure 2). This is consistent with previous publications with similar microinjection sites (Blandini et al., 2009; Decressac et al., 2012; Gambaryan et al., 2014; Pellegrini et al., 2017). Out of 68 rats microinjected with 6-OHDA for all studies, two had no reduction of TH, indicative of misplacement of the injection site and were excluded of data analysis.

**Figure 3:**
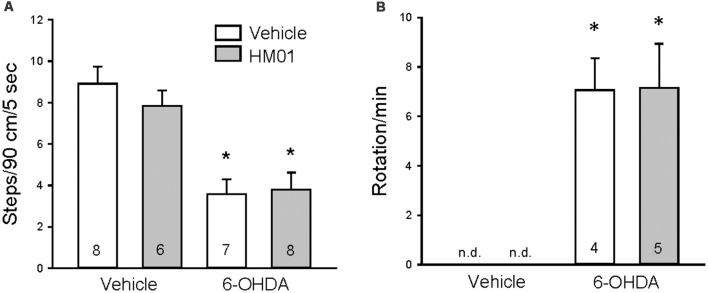
Repeated treatments with ghrelin agonist, HM01 did not modify motor impairments in 6-OHDA rat Parkinson’s disease (PD) model. Rats were microinjected with 6-OHDA or vehicle unilaterally into the medial forebrain bundle. HM01 (3 mg/kg) or vehicle was administered og daily for 12 days starting from 4 weeks after microinjection. Stepping **(A)** and apomorphine-induced rotation **(B)** tests were performed on the last 2 days at 4–6 h after HM01 treatment in vehicle and 6-OHDA rats. Data are mean ± standard error of the mean (SEM) and animal numbers per group indicated in the graphs. *p < 0.05 vs. vehicle/vehicle by one-way analysis of variance (ANOVA).

The 6-OHDA rats showed forelimb akinesia in the stepping test as indicated by the significant decrease in adjusted steps compared to vehicle microinjected rats (4.5 ± 0.6 vs. 9.4 ± 0.5 steps/90 cm/5 s, *p* < 0.05, *n* = 14–15). After 12 days of og vehicle treatment, adjusted steps by 6-OHDA rats were still significantly lower than controls (3.6 ± 0.7 vs. 8.9 ± 0.8 steps/90 cm/5 s, *p* < 0.05, Figure 3A). Chronic daily HM01 in 6-OHDA rats did not improve the stepping performance compared HM01/vehicle group (3.8 ± 0.8 vs. 7.8 ± 0.7 steps/90 cm/5 s, *p* < 0.05, Figure 3A). In the rotation test (Figure 3B), rats with unilateral lesion of SN display rotations after the ip injection of apomorphine. Vehicle/vehicle and HM01/vehicle rats did not show the occurrence of rotation. HM01 treatment in 6-OHDA rats did not reduce the apomorphine-induced rotations compared to vehicle/6-OHDA rats (7.1 ± 1.3 vs. 7.2 ± 1.8 turns/min, *p* > 0.05).

### Changes in Water Intake, Food Consumption and Fecal Output in 6-OHDA Rats

**Figure 4:**
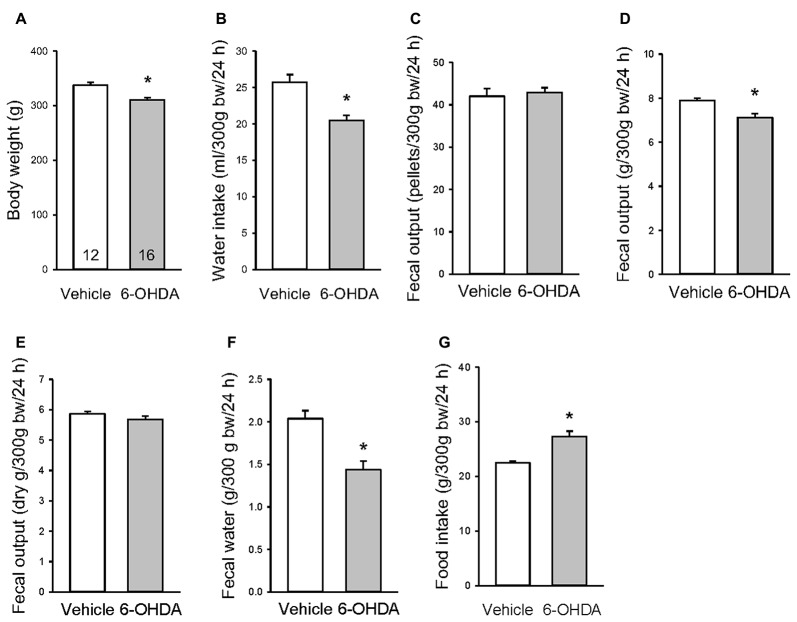
Basal body weight **(A)**, 24-h water intake **(B)**, fecal pellets **(C)**, fecal weight **(D)**, dry fecal weight **(E)**, fecal water content **(F)** and food intake **(G)** in rats microinjected with 6-OHDA or vehicle unilaterally into the medial forebrain bundle. The assessments were performed in 2–3 days between 3–4 weeks after the microinjection. Data are mean ± SEM, calculated per 300 g body weight (bw). Number of rats/group indicated in each bar of graph A. *p < 0.05 vs. vehicle by Student *t*-test.

Under basal conditions examined 3 weeks after the mfb microinjection, 6-OHDA rats had a significant 8% reduction of their body weight (Figure 4A), and 20%, 10% and 30% decrease in 24-h water intake, fecal output weight and water content respectively compared with vehicle microinjected rats (Figures 4B,D,F). There were no differences in fecal pellet numbers and dried fecal weight per 24 h (Figures 4C,E). The food amount measured for 24 h showed a significant increase between 6-OHDA and vehicle rats (Figure 4G).

**Figure 5:**
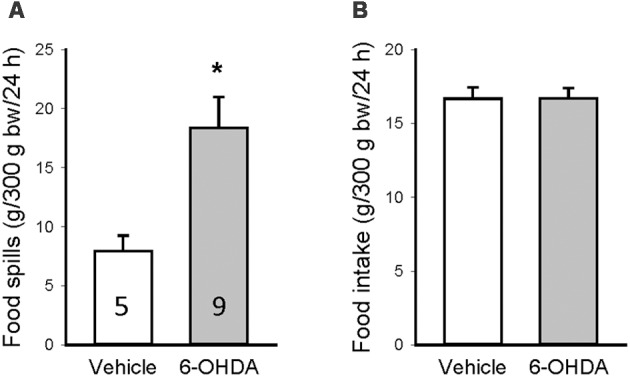
Food spillage assessed 3 weeks after brain microinjection when the rats were housed in cages with a grid to separate the food spills. Food spills **(A)** and food intake **(B)** were measured for 24 h and calculated per 300 g body weight (bw). Data are mean ± SEM and animal numbers per group indicated in the graphs. *p < 0.05 vs. vehicle/vehicle by Student *t*-test.

To assess whether the increase in food intake could reflect spills caused by motor impairments, 3 weeks after unilateral microinjection in the mfb, another group of rats was monitored for basal food intake when housed in cages with a grid at the bottom to separate the spills. The amount of food spills during 24 h was significantly higher in 6-OHDA than vehicle rats (18.4 ± 2.6 vs. 7.9 ± 1.3 g/24 h, Figure 5A), whereas the food intake was the same between the two groups (16.7 ± 0.8 vs. 16.7 ± 0.7 g/24 h, Figure 5B). The difference between 6-OHDA and vehicle rats was bigger than the rats housed in normal housing due to the dropping of food pieces through the grid by 6-OHDA rats with motor impairment.

### Chronic Daily HM01 Treatments Normalized Defecation and Increased Drinking and Feeding in 6-OHDA Rats

**Figure 6:**
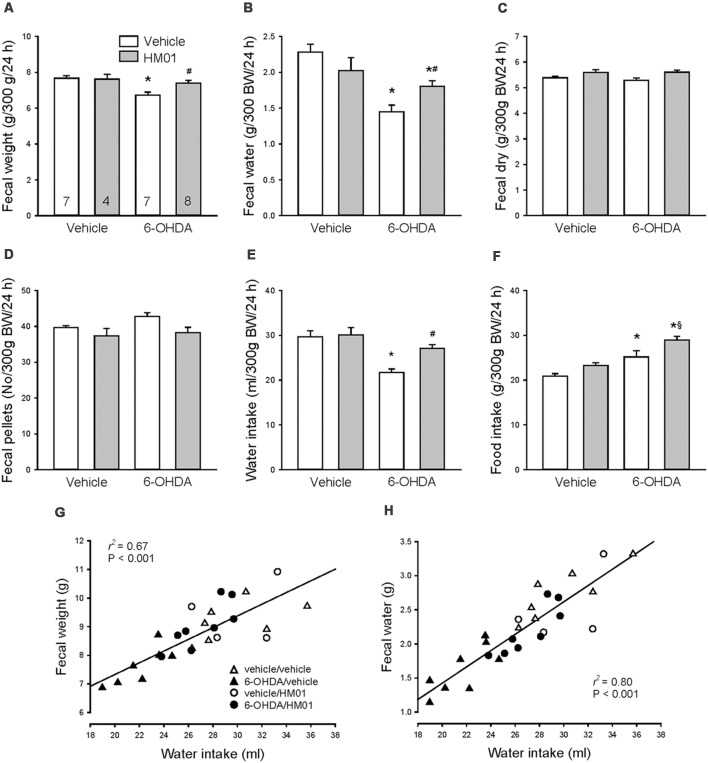
Effect of orogastric daily administration of HM01 (3 mg/kg) for 10 days on average daily fecal weight **(A)**, fecal water content **(B)**, fecal dry weight **(C)**, fecal pellets **(D)**, and intake of water **(E)** and food **(F)** in rats microinjected with 6-OHDA or vehicle unilaterally into the medial forebrain bundle (3–4 weeks before). Data are mean ± SEM calculated per 300 g of body weight (bw). Rats/group are indicated in each bar of graph **(A)**. *p* < 0.05 as *: vs. vehicle/vehicle, #: vs. 6-OHDA/vehicle and §: vs. vehicle/HM01 by one-way and two-way ANOVA. Correlation of the average 24-h water intake with fecal weight **(G)** and with fecal water content **(H)** during 10 days treatment of HM01 (og at 3 mg/kg) analyzed by linear regression.

As observed under basal conditions, 10 days after og vehicle, the 6-OHDA rats had a significant 13%, 37% and 21% reduction of fecal weight, fecal water and water intake/24 h respectively and 21% increase in food intake compared with vehicle/vehicle group (Figures 6A,B,E). Treatment with HM01 (3 mg/kg, og) once a day for 10 days had no significant effect on those parameters in mfb vehicle rats compared with vehicle/vehicle group. However, in 6-OHDA rats, the HM01 treatment normalized the reduced fecal weight (Figure 6A) to values of vehicle/vehicle group. The ghrelin agonist treatment significantly increased fecal water content and water intake, although values are still significantly lower than those of vehicle/vehicle group (Figures 6B,E). HM01 also increased food intake (Figure 6F) compared to 6-OHDA/vehicle group. The pellet numbers and dry weight were not different among the four treatment groups (Figures 6C,D). Two-way ANOVA showed the influence of 6-OHDA in food intake (*F*_(1,22)_ = 23.40, *p* < 0.001), water intake (*F*_(1,22)_ = 14.83, *p* < 0.001), fecal weight (*F*_(1,22)_ = 10.76, *p* < 0.01) and fecal water content (*F*_(1,22)_ = 23.01, *p* < 0.001); and HM01 in food intake (*F*_(1,22)_ = 8.89, *p* < 0.01), fecal dry weight (*F*_(1,22)_ = 7.85, *p* = 0.01) and fecal pellet numbers (*F*_(1,22)_ = 6.12, *p* < 0.05). The interaction between 6-OHDA and HM01 showed significance in fecal water content (*F*_(1,22)_ = 7.80, *p* = 0.01), while not in other parameters.

Daily fecal output weight and water content were highly correlated with water intake (*r*^2^ = 0.67 and *r*^2^ = 0.80, *p* < 0.001 respectively; Figures 6G,H), whereas fecal output weight was not correlated with food intake (*r*^2^ = 0.13, *p* > 0.05), and neither food intake with water intake (*r*^2^ = 0.01, *p* > 0.05).

Time course of 24-h measurements at each day during HM01 treatments showed a significant plateau increase of fecal weight, fecal water content and water intake occurring the 1st day that was maintained throughout the 10 days experimental period (Table 1). Likewise, there was no significant difference between the first and last day of 24-h food intake measurement although the days 2–5 had significant daily higher intake than days 7, 9 and 10 (Table 1).

**Table 1:** No desensitization of HM01 effect in 6-OHDA rats during the treatment.

Day	0*	1	2	3	4	5	6	7	8	9	10	
Fecal weight	6.9 ± 0.3	7.9 ± 0.2	8.3 ± 0.1	7.9 ± 0.2	8.0 ± 0.4	8.0 ± 0.3	7.4 ± 0.3	7.5 ± 0.3	7.6 ± 0.3	7.4 ± 0.4	7.7 ± 0.3	*F* = 1.16, *p* > 0.05
Fecal water	1.4 ± 0.1	1.7 ± 0.1	2.0 ± 0.1	1.9 ± 0.1	2.0 ± 0.2	2.1 ± 0.2	1.7 ± 0.2	1.8 ± 0.2	1.9 ± 0.2	1.9 ± 0.2	2.1 ± 0.1	*F* = 1.16, *p* > 0.05
Water intake	19.7 ± 0.7	24.5 ± 0.7	24.0 ± 1.2	23.7 ± 0.7	24.4 ± 0.5	24.0 ± 0.8	23.5 ± 0.6	22.0 ± 1.0	23.4 ± 0.9	21.4 ± 0.3	23.2 ± 0.9	*F* = 1.83, *p* > 0.05
Food intake	25.2 ± 2	30.6 ± 0.9	32.5 ± 1.5	32.4 ± 1.2	32.9 ± 1.0	32.7 ± 1.6	30.0 ± 1.3	28.4 ± 1.2	29.1 ± 0.9	27.1 ± 1.1	28.4 ± 1.5	*F* = 7.45, *p* <0.05^§^

Data are means/24 h ± SEM, *n* = 8, analyzed by one-way repeated measures analysis of variance. 0*: measurements in 6-OHDA rats before HM01 treatment, *p* < 0.05 vs. days with HM01. ^§^: significant as 24-h food intake on D2–D5 compared with Day 7, 9 or 10, but not with Day 1, and not D1 vs. D10.

### HM01 Increased Body Weight and Fat Gain in 6-OHDA Rats

**Figure 7:**
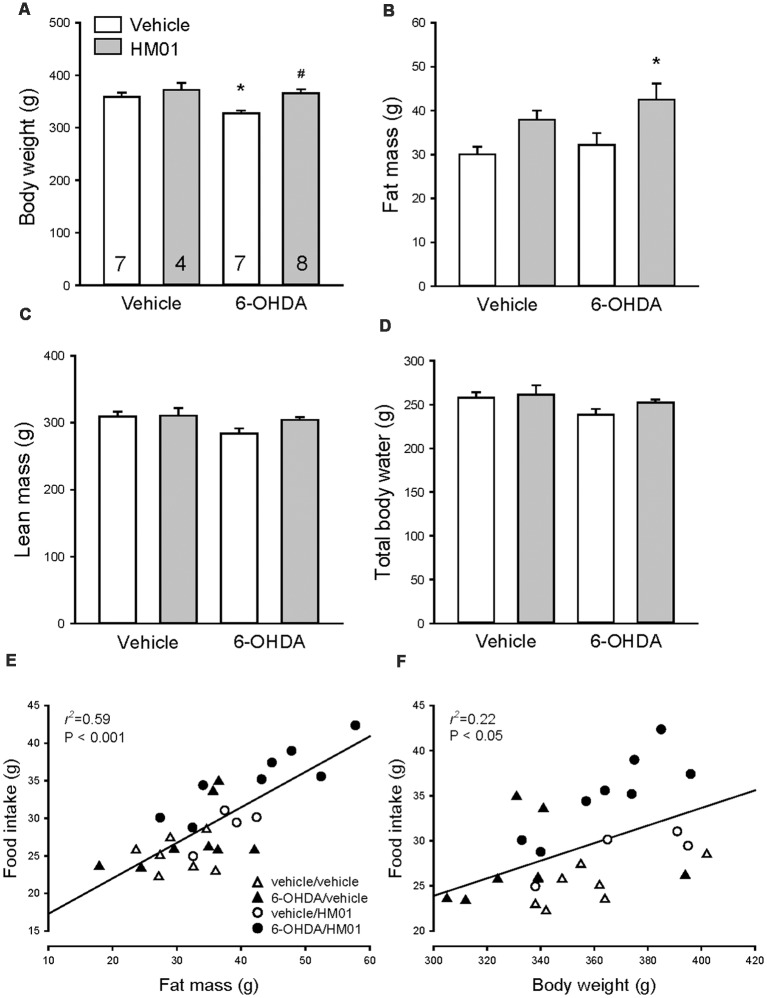
Body weight and composition of rats microinjected with 6-OHDA or vehicle unilaterally into the medial forebrain bundle after HM01 or vehicle treatments. HM01 (3 mg/kg) or vehicle was administered og daily for 10–12 days starting from 3 to 4 weeks after microinjection of 6-OHDA. **(A)** Body weight; **(B)** fat mass; **(C)** lean mass; **(D)** total body water. Body fat mass **(E)** and body weight **(F)** are correlated with food intake. Data are mean ± SEM. Rats/group are indicated in each bar of graph **(A)**. *p* < 0.05 as *: vs. vehicle/vehicle, and #: vs. 6-OHDA/vehicle by one-way ANOVA. Two-way ANOVA revealed an interaction between 6-OHDA and HM01 on changes of body weight, lean mass and total body water (*p* > 0.05), and the HM01 increased body fat mass (*p* < 0.05).

Before HM01 treatment 6-HODA rats had a significantly lower body weight compared to vehicle rats (321.9 ± 4.3 g, *n* = 15, vs. 346.7 ± 5.6 g, *n* = 11, *p* < 0.001) while showing no significant changes in fat mass and a non-significant decrease in lean mass and body total water. In mfb-vehicle rats, HM01 (3 mg/kg, og) administration for 10–12 days had no significant effect on body weight, lean mass and body water (Figures 7A,C,D) and there is a trend to increase fat mass (Figure 7B). HM01 treatment in 6-OHDA rats normalized the decreased body weight values to those of vehicle/vehicle controls (Figure 7A) and significantly increased the fat mass (Figure 7B) while there was a non-significant increase in lean mass or body water (Figures 7C,D). Two-way ANOVA revealed influences of 6-OHDA and HM01 in body weight (*F*_(1,22)_ = 5.21 and 9.57, *p* < 0.05), lean mass (*F*_(1,22)_ = 9.48 and 5.29, *p* < 0.05) and total body water (*F*_(1,22)_ = 9.98 and 4.63, *p* < 0.05), and interaction of 6-OHDA and HM01 on lean mass (*F*_(1,22)_ = 4.43, *p* < 0.05), whereas HM01 only significantly influenced the body fat mass (*F*_(1,22)_ = 8.48, *p* < 0.05).

Body fat mass was significantly correlated with food intake (*r*^2^ = 0.59, *p* < 0.001), and to a small extent with body weight (*r*^2^ = 0.22, *p* < 0.05; Figures 7E,F).

## Discussion

This study provides new insight into functional alterations occurring in the neurotoxin PD model with unilateral lesion of the nigrostriatal system induced by 6-OHDA microinjected unilaterally into the mfb. We found that the reduced daily fecal weight and water content were correlated with the reduction of water intake. More importantly, we demonstrated the beneficial effects of repeated daily oral treatment with the long-acting ghrelin agonist, HM01 (Karasawa et al., 2014a) on some non-motor alterations namely the constipation-like defecation, reduction of water intake and body weight. The HM01-increased body weight gain could result from increased energy intake, lean mass and more prominently fat mass. However, HM01 did not modify 6-OHDA-induced motor impairments, indicating that the ghrelin agonist exerts its actions primarily by influencing altered feeding and drinking behavior.

The 24 h fecal water content is highly correlated to water intake in 6-OHDA rats treated with and without HM01. This finding is consistent with two clinical reports showing a reduction of liquid intake in PD patients associated with the incidence of constipation (Ueki and Otsuka, 2004; Gan et al., 2018). In addition, experimental evidence demonstrated that DA pathways from the midbrain to forebrain limbic system play a role in anticipatory and motivated drinking behavior (Cone et al., 2016; Hsu et al., 2018). Activation of DA signals in the striatal system increased water intake (Pal et al., 1992; Amato et al., 2012), while lesion by 6-OHDA attenuated angiotensin II-reduced drinking in rats (Sumners et al., 1981). The high correlation of water intake and fecal water content suggests that the constipation-like defecation is mainly a consequence of reduction of water intake, which may be specific to this model that has decreased DA signals in the nigrostriatal system. The 6-OHDA rats may be a relevant model to delineate the role of DA signaling and interactions with other circuits that regulate drinking behavior. In addition, the decrease in water intake, unlike food consumption in 6-OHDA rats, points to possible disassociated central mechanisms underlying altered food and water intakes in rats with 6-OHDA-induced loss of dopaminergic neurons, as found in other experimental conditions (Karasawa et al., 2014b; Zimmerman et al., 2017; Gizowski and Bourque, 2018).

Our data further showed that HM01 partially resumed reduced water intake and fecal water content in 6-OHDA rats, and further increased food intake in 6-OHDA rats, while having no significant effects in controls. HM01 effect on water intake could be related to the activation of ghrelin receptors in reward center, as reports showed that ghrelin or agonist injected into the VTA induced reward behaviors (Perelló and Zigman, 2012; Stievenard et al., 2017). Although ghrelin inhibition of water intake was previously reported in rats, the study was done under conditions of dehydration-induced drinking such as 24 h water deprivation or administration of angiotensin II (Hashimoto and Ueta, 2011). Further studies are needed to delineate whether 6-OHDA rats have alterations of fluid homeostasis and lower drive for drinking and to investigate the central circuitry that causes decreased water intake and mediates ghrelin effect on drinking behavior.

Rats with 6-OHDA lesion in the nigrostriatal system weigh less than control rats, indicating that the loss of dopaminergic neurons in the nigrostriatal system could play a role in maintaining body weight. Weight loss is common in PD patients and more prominent with the progression of the disease. It is associated with fat mass loss (Lorefält et al., 2004; Pålhagen et al., 2005) and caused by heterogenic factors including malnutrition (Mukherjee et al., 2016; Sharma and Lewis, 2017). However, there are also reports on weight gain in PD patients at the early stage (Vikdahl et al., 2014). In the present study, 6-OHDA rats did not lose body fat while they weighed less than vehicle controls. The reduction in body weight in 6-OHDA rats could be associated with the combined trends of lower lean mass and body water content. By contrast, the basal food intake was increased when monitored 3 weeks after the brain microinjection. This change may reflect the component of the increased spills because of motor deficits of the 6-OHDA rats. This is supported by the food spilling control experiment using a grid to separate the spills. We showed the 6-OHDA rats ate the same amount of food as vehicle controls while the use of the grid resulted in more small pieces of chow dropped below the grid in 6-OHDA than vehicle rats. It is to note that the spillage was much more than that in normal housing conditions. Thus, we did not perform tests with HM01 using the same setting for all other experiments.

HM01 significantly increased 24-h food intake in 6-OHDA rats while having no significant effect on that in vehicle microinjected rats. HM01 also increased body weight gain in 6-OHDA rats with a prominent increase in body fat mass, which was correlated to food intake, indicative of positive energy intake. HM01 stimulated feeding possibly involves brain pathways since HM01 is brain penetrant and activates neurons in nuclei involved in food regulation, the hypothalamic arcuate nucleus and nucleus tractus solitarius (Karasawa et al., 2014a). Ghrelin acts centrally to induce adiposity (Wren et al., 2001; Theander-Carrillo et al., 2006; Al Massadi et al., 2017) as well as peripherally to increase lipogenesis (Müller and Tschöp, 2013; Müller et al., 2015). As HM01 crosses the blood brain barrier, it may act both in the brain and in the periphery to increase fat mass. The ability of ghrelin agonist to increase body weight, fat mass and food intake should be beneficial to PD patients who lose body weight due to malnutrition, especially in the late stage when patients have a more prominent loss of fat mass (Mukherjee et al., 2016).

Ghrelin prokinetic effect on GI motility involves the central nervous system (Poitras and Tomasetto, 2009; Sallam and Chen, 2010). We demonstrated in our previous study that oral administration of HM01 increased c-Fos expression in the lumbosacral spinal intermediolateral column and arcuate nucleus in the hypothalamus in 6-OHDA rats (Karasawa et al., 2014a), which bear the central mechanisms regulating colonic functions (Tebbe et al., 2005; Hirayama et al., 2010; Ferens et al., 2011). In support of this contention, studies showed that ghrelin acted in the hypothalamus to stimulate GI motility (Wang et al., 2015; Huang et al., 2017), and ghrelin and its agonists improved defecation in rats primarily through lumbo-sacral spinal component (Hirayama et al., 2010; Pustovit et al., 2014, 2015). Studies should be directed to unveil brain circuity linked to the mesolimbic pathways for ghrelin agonist modulatory effect on the GI propulsive motor function (Anselmi et al., 2017; Garrido-Gil et al., 2018).

In conclusion, our data further demonstrated that 6-OHDA-induced loss of nigrostriatal DA neurons decreased fecal weight and water content, which is correlated to a decrease in water intake. Oral chronic HM01 treatment did not alleviate motor impairment induced by the 6-OHDA while normalizing the reduction of body weight, constipation-like feature most likely by increasing water and food intake and fecal water content, beside the well-known prokinetic effect on the GI tract (Poitras and Tomasetto, 2009; Greenwood-Van Meerveld et al., 2011; Mosińska et al., 2017). The use of orally active, long-acting ghrelin agonist may be a promising venue to alleviate non-motor PD symptoms related to energy balance in those patients with decreased water and food intakes, delayed GI propagation, and reduced body mass. These beneficial effects will be important to improve patients’ quality of life (Mosińska et al., 2017).

## Ethics Statement

Animal care and experimental procedures followed institutional ethic guidelines and conformed to the requirements of federal regulations for animal research conduct. All procedures were approved by the Animal Research Committee at Veterans Affairs Greater Los Angeles Healthcare System (animal protocol #01001-15).

## Author Contributions

AM and LG: microinjections in rats, *in vivo* experiments. CP: HM01 production and reviewed the manuscript. YT: experimental design and contributed to the writing of the manuscript. LW: study design, *in vivo* experiments performance, analysis and wrote the manuscript.

## Conflict of Interest Statement

CP is employed by Helsinn SA Lugano, in Lugano, Switzerland. The remaining authors declare that the research was conducted in the absence of any commercial or financial relationships that could be construed as a potential conflict of interest.

## Abbreviations

6-OHDA: 6-hydroxydopamine
CMC: carboxymethyl cellulose
DA: dopamine or dopaminergic
GI: gastrointestinal
GHSR: growth hormone secretagogue receptor
mfb: medial forebrain bundle
MRI: magnetic resonance imaging
og: orogastric or orogastrically
PD: Parkinson’s disease
SN: substantia nigra
TH: tyrosine hydroxylase.
